# Antigen 43 associated with *Escherichia coli* membrane vesicles contributes to bacterial cell association and biofilm formation

**DOI:** 10.1128/spectrum.01890-24

**Published:** 2025-01-22

**Authors:** Lauren Zavan, Lilian Hor, Ella L. Johnston, Jason Paxman, Begoña Heras, Maria Kaparakis‑Liaskos

**Affiliations:** 1 Department of Microbiology, Anatomy, Physiology, and Pharmacology, La Trobe University, Melbourne, Victoria, Australia; 2 Department of Microbiology and Immunology, The Peter Doherty Institute for Infection and Immunity, University of Melbourne, Melbourne, Victoria, Australia; 3 Department of Biochemistry and Chemistry, La Trobe Institute for Molecular Sciences, La Trobe University, Melbourne, Victoria, Australia; Griffith University-Gold Coast Campus, Gold Coast, Queensland, Australia

**Keywords:** bacterial membrane vesicles, outer membrane vesicles, autotransporters, biofilm, Antigen 43

## Abstract

Bacterial membrane vesicles (MVs) are produced by all bacteria and contribute to numerous bacterial functions due to their ability to package and transfer bacterial cargo. In doing so, MVs have been shown to facilitate horizontal gene transfer, mediate antimicrobial activity, and promote biofilm formation. Uropathogenic *Escherichia coli* is a pathogenic Gram-negative organism that persists in the urinary tract of its host due to its ability to form persistent, antibiotic-resistant biofilms. The formation of these biofilms is dependent upon proteins such as Antigen 43 (Ag43), which belongs to the widespread Autotransporter group of bacterial surface proteins. In *E. coli,* the autotransporter Ag43 has been shown to contribute to bacterial cell aggregation and biofilm formation via self-association of Ag43 between neighboring Ag43-expressing bacteria. As MVs package bacterial proteins, we investigated whether MVs produced by *E. coli* contained Ag43, and the ability of Ag43-expressing MVs to facilitate cell aggregation and biofilm formation. We showed that Ag43 expressing *E. coli* produced MVs that contained Ag43 on their surface and had an enhanced ability to bind to *E. coli* bacteria. Furthermore, we demonstrated that the addition of Ag43-containing MVs to Ag43-expressing *E. coli* significantly enhanced biofilm formation. These findings reveal the contribution of MVs harboring autotransporters in promoting bacterial aggregation and enhancing biofilm formation, highlighting the impact of MVs and their specific composition to bacterial adaptation and pathogenesis.

**IMPORTANCE:**

Autotransporter proteins are the largest family of outer membrane and secreted proteins in Gram-negative bacteria which contribute to pathogenesis by promoting aggregation, biofilm formation, persistence, and cytotoxicity. Although the roles of bacterial autotransporters are well known, the ability of bacterial membrane vesicles (MVs) naturally released from the surface of bacteria to contain autotransporters and their role in promoting virulence remains less investigated. Our findings reveal that MVs produced by *E. coli* contain the autotransporter protein Ag43. Furthermore, we show that Ag43-containing MVs function to enhance bacterial cell interactions and biofilm formation. By demonstrating the ability of MVs to carry functional autotransporter adhesins, this work highlights the importance of MVs in disseminating autotransporters beyond the bacterial cell membrane to ultimately promote cellular interactions and enhance biofilm development. Overall, these findings have significant implications in furthering our understanding of the numerous ways in which MVs can facilitate bacterial persistence and pathogenesis.

## INTRODUCTION

Bacterial membrane vesicles (MVs) are nanoparticles produced by bacteria as part of their normal growth that contain a range of bacterial cargo, including nucleic acids and proteins derived from their parent bacteria (1, 2). Due to their biological load, MVs can function as an extracellular delivery system of bacterial proteins and nucleic acids to bacteria or host epithelial cells to promote pathogenesis (3–6), mediate inflammation (7–10), or enhance bacterial survival and biofilm formation (11–13). Recent findings reveal that Gram-negative bacteria produce numerous subtypes of MVs, consisting of MVs that solely contain the bacterial outer membrane or MVs that contain both outer and inner bacterial membranes (1). As a result, MVs derived from Gram-negative bacteria are often enriched in numerous outer membrane proteins (14–17). However, the ability of MVs to package functional outer membrane proteins that facilitate bacterial adhesion and biofilm formation is not well established.

One of the largest families of secreted, and outer membrane proteins in Gram-negative bacteria are the autotransporter proteins, which contribute to a number of bacterial functions including adhesion, invasion, toxicity, and biofilm formation (18–20). In *E. coli*, the major group of autotransporter proteins are the self-associating autotransporters (SAATs) that are also known as adhesin involved in diffuse adherence (AIDA-I) type proteins (21), and of these, Antigen 43 (Ag43) is the most prevalent (22). Ag43 is a cell-surface-associated autotransporter that mediates cell-to-cell aggregation and biofilm formation (23–25) and is expressed by both pathogenic and commensal *E. coli* strains (26). Similar to other autotransporter proteins, Ag43 possesses a segmented domain organization with an N-terminal signal sequence, that grants secretion of the protein across the inner membrane, and a C-terminal translocator (β43) domain which facilitates the translocation of the central functional α-domain (α43) to the cell surface (27–30). Although the functional α43 domain is processed after translocation, it remains bound to the translocating β43 domain and is displayed on the bacterial surface to promote phenotypes such as bacterial aggregation and biofilm formation (31–35). Structural investigation of Ag43 from uropathogenic *E. coli* identified that the passenger domain promotes aggregation and biofilm formation, by forming head-to-tail self-associations between α43a molecules on the surface of neighboring *E. coli* cells in a “molecular Velcro-type mechanism” (36). Examination of Ag43 homologs from diverse pathogenic *E. coli* strains revealed this is a common mode of self-association among Ag43 proteins that drives bacterial aggregation (37) and allows for Ag43 proteins from different *E. coli* strains to associate and drive aggregation and biofilm formation (38).

Despite the key role of autotransporters such as Ag43 in biofilm formation, the ability of MVs to display functional autotransporters that may contribute to promoting their phenotypes has not been elucidated. In this study, we aimed to determine whether Ag43-expressing *E. coli* produced MVs containing functional Ag43, and the ability of Ag43-containing MVs to contribute to aggregation and biofilm formation. To understand this, we isolated and characterized MVs produced by both Ag43-expressing (Ag43^+ve^) and Ag43-null (Ag43^−ve^) *E. coli* to determine the effect of Ag43 expression on MV release. We confirmed the presence of Ag43 on *E. coli* MVs produced during normal growth conditions, and demonstrated that Ag43^+ve^ MVs adhered to *E. coli* bacteria in an Ag43-dependent manner to ultimately enhance biofilm formation. This work reveals the contribution of Ag43^+ve^ MVs to promoting *E. coli* biofilm formation and identifies a novel role of MV-associated autotransporters in enhancing MV-bacterial interactions and biofilm development.

## RESULTS

### *E. coli* expressing Antigen 43 have increased MV production and a reduction in MV size

To examine if *E. coli* MVs contained functional Ag43, we used the parental *E. coli* strain HEHA16, a *Δfim::kan Δagn43* derivative of *E. coli* BD1428, lacking both type 1 fimbriae and Ag43 (34). To produce Ag43-expressing or Ag43-null MVs, *E. coli* HEHA16 that either harbored the pBAD/MycHisA::*agn43* plasmid, encoding for full-length Ag43 that could be overexpressed with the addition of L-arabinose- (Ag43^+ve^ *E. coli*) or an empty pBAD/MycHisA plasmid vector (Ag43^−ve^ *E. coli*), were used in this study. The use of an *E. coli* strain without type 1 fimbriae is important for studies of Ag43, as previous work has shown that type I fimbrial expression represses Ag43 transcription (39) and abolishes Ag43 self-aggregation (33). This choice of bacterial strain and plasmid allowed us to control Ag43 expression while avoiding the regulatory complexities of endogenous type 1 fimbriae and Ag43. Previous research has demonstrated that these two adhesins undergo phase variation and are inversely regulated during infection, making it challenging to investigate Ag43-specific effects in a native context. By using a strain lacking both adhesins and introducing Ag43 via the pBAD/MycHisA plasmid, we could mimic Ag43-expressing infection conditions and approximate the high expression levels of endogenous Ag43, which have been estimated to reach up to 50,000 copies per cell (22, 40).

**Fig 1:**
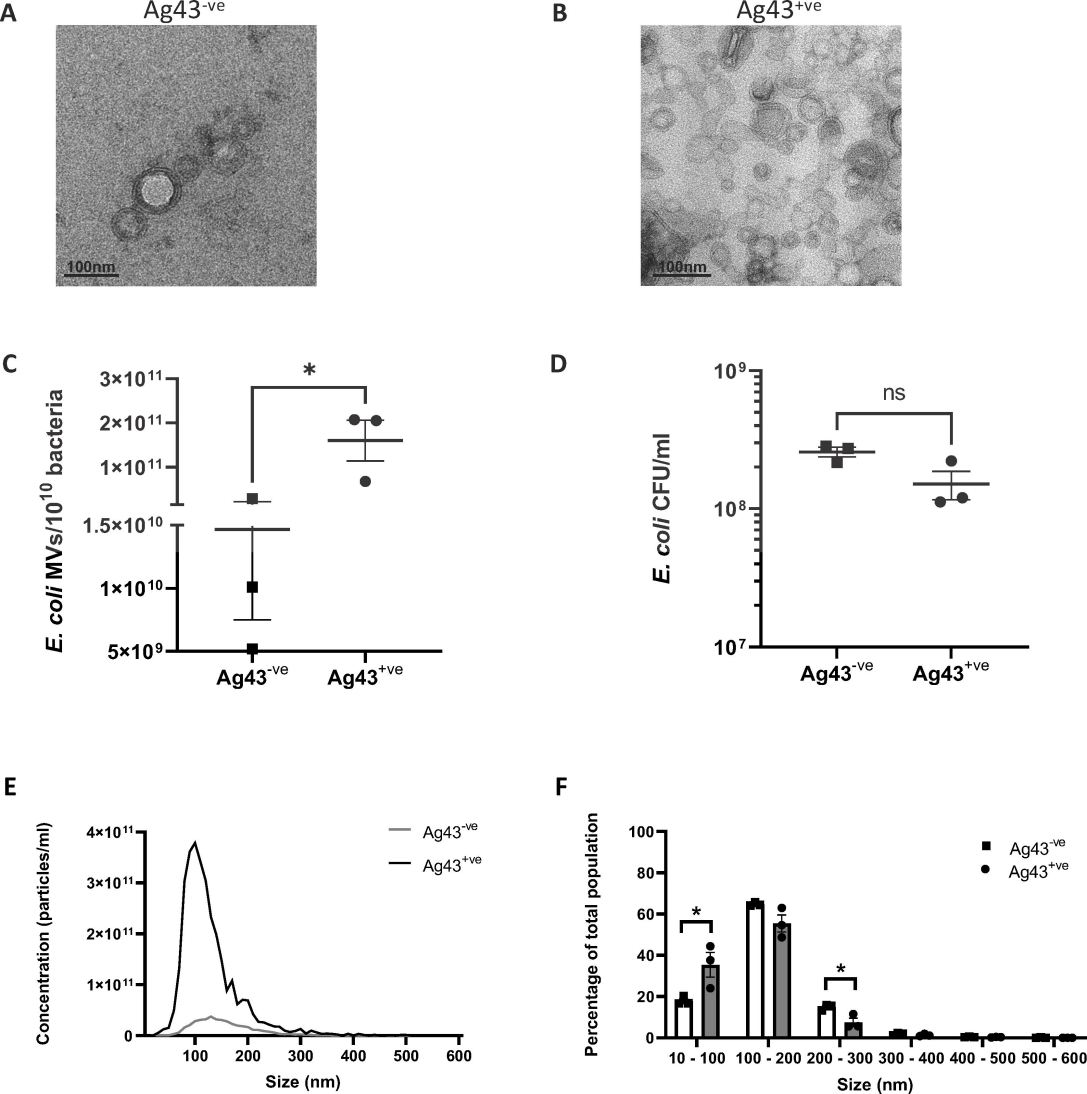
Characterization of *E. coli* Ag43^−ve^ and Ag43^+ve^ MVs. MVs isolated from (**A**) *E. coli* HEHA16 pBAD/MycHisA (Ag43^−ve^) and (**B**) *E. coli* HEHA16 pBAD/MycHisA::*agn43* (Ag43^+ve^) were visualized by transmission electron microscopy. Scale bar = 100 nm. Images are representative of three biological replicates. (**C**) The number of Ag43^−ve^ and Ag43^+ve^ MVs produced per 10^10^ bacterial cells was determined by ZetaView Nanoparticle Tracking Analysis (NTA). Data are represented as the mean ± SEM of three biological replicates. *P < 0.05, unpaired *t*-test. (**D**) The number of viable Ag43^−ve^ and Ag43^+ve^ *E. coli* HEHA16 bacteria present in individual cultures from which MVs were isolated was determined by viable counts. Data show CFU/mL of individual cultures, and the mean ± SEM of three biological replicates. ns, not significant. (**E**) The size distribution of Ag43^−ve^ MVs and Ag43^+ve^ MVs was determined using ZetaView NTA. Data represent the mean of three biological replicates. (**F**) Quantification of the size distribution of Ag43^−ve^ MVs (squares; open bars) or Ag43^+ve^ MVs (circles; grey bars) by ZetaView NTA. Data are represented as the mean ± SEM of three biological replicates. *P < 0.05, unpaired *t*-test within each size range.

MVs isolated from Ag43^−ve^ and Ag43^+ve^ *E. coli* were purified using density gradient ultracentrifugation, and their morphology and quantity were determined using transmission electron microscopy (TEM) and Nanoparticle Tracking Analysis (NTA), respectively. Both Ag43^−ve^ MVs and Ag43^+ve^ MVs were heterogenous in size and displayed a similar spherical MV morphology (Fig. 1A and B). Quantification of the number of MVs produced per 10^10^ bacteria at the time of MV isolation demonstrated that Ag43^+ve^ *E. coli* produced significantly more MVs compared to Ag43^−ve^ *E. coli* (*P* < 0.05; Fig. 1C). The increase in MV production by Ag43^+ve^ *E. coli* was not due to enhanced bacterial growth, as there was no significant difference in the number of bacteria present in Ag43^+ve^ and Ag43^−ve^ *E. coli* cultures at the time of MV collection (Fig. 1D), suggesting that enhanced MV production by Ag43^+ve^ *E. coli* was attributed to the overexpression of Ag43.

Next, we examined if Ag43 expression by *E. coli* affected the size of MVs produced. Analysis of MV size by NTA revealed that Ag43^−ve^ MVs ranged from 50 to 400 nm in size with the predominant population of MVs being 110 nm in diameter (Fig. 1E). Comparatively, Ag43^+ve^ MVs ranged between approximately 50 and 300 nm in size with the majority of MVs being approximately 90 nm in size (Fig. 1E). Further examination revealed that there were significant differences in the size of MVs produced by Ag43^+ve^ and Ag43^−ve^ *E. coli* (Fig. 1F). Specifically, there was a significant increase in the proportion of MVs ranging from 10 to 100 nm in diameter produced by Ag43^+ve^ bacteria compared to MVs produced by Ag43^−ve^ bacteria (*P* < 0.05; Fig. 1F), and a smaller percentage of Ag43^+ve^ MVs ranging between 200 and 300 nm in size compared to Ag43^−ve^ MVs (*P* < 0.05; Fig. 1F). Overall, Ag43 expressing *E. coli* produced significantly more MVs, that contained a larger proportion of smaller MVs, compared to MVs produced by Ag43^−ve^ *E. coli*.

### *E. coli* MVs contain bacterial cargo including Antigen 43

**Fig 2:**
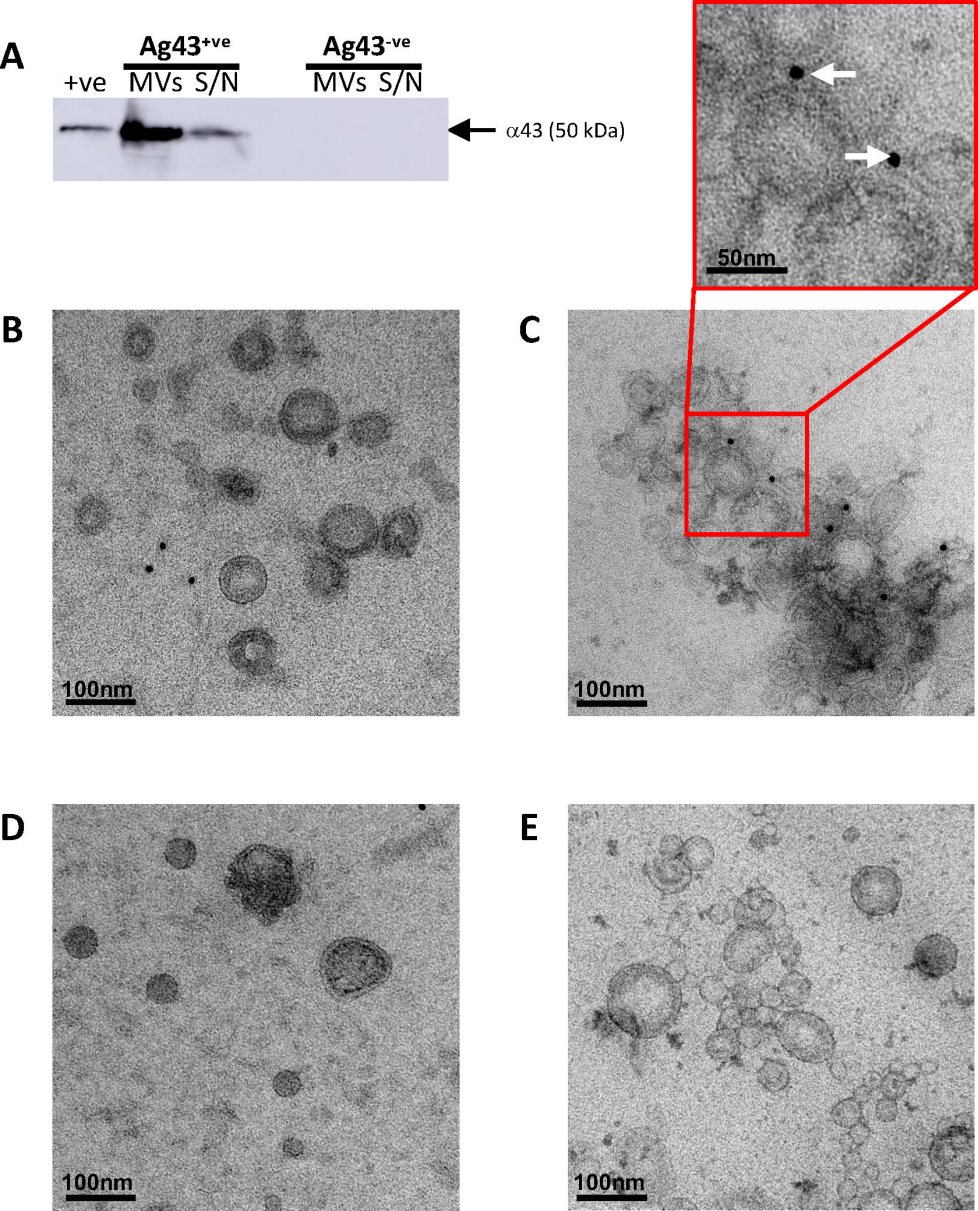
*E. coli* MVs contain Antigen 43. (**A**) MVs and MV-depleted supernatants (S/N) obtained from Ag43^+ve^ *E. coli* and Ag43^−ve^ *E. coli* cultures were separated using SDS-PAGE, and the presence of Ag43 was detected by Western blot using an anti-Ag43 antibody. Arrow indicates α43 (50 kDa) and purified Ag43 was used as a control (+ve). The image is representative of three biological replicates. (**B**) *E. coli* Ag43^−ve^ MVs and (**C**) Ag43^+ve^ MVs were labeled with rabbit anti-Ag43 antibodies, followed by an anti-rabbit antibody and protein-A-gold (PAG) and imaged by TEM. White arrows within the magnified image indicate the association of PAG with Ag43^+ve^ MVs. (**D**) Ag43^−ve^ MVs and (**E**) Ag43^+ve^ MVs were labeled with anti-rabbit antibodies and protein-A-gold (PAG) as a negative control. Images are representative of three biological replicates. Scale bar = 100 nm for all images and 50 nm in the magnified image.

As the association of Ag43 with MVs has only been identified via proteomic analysis of either crude, non-purified, or detergent-derived *E. coli* MVs (41, 42), the presence of Ag43 on a population of naturally released and purified *E. coli* MV preparation has not yet been shown. Therefore, we sought to determine if Ag43 was associated with *E. coli* MVs that were released naturally by *E. coli* as part of their normal growth. Density gradient purified Ag43^−ve^ and Ag43^+ve^ *E. coli* MVs were separated by SDS-PAGE, and the presence of Ag43 in MVs was determined by Western blot using an anti-Ag43 antibody that detects the extracellular functional α-subunit-component of Ag43 (α43) (36). We detected a 50 kDa protein in Ag43^+ve^ *E. coli* MVs that corresponded to the expected molecular weight of α43 (36, 40) (Fig. 2A). As expected, Ag43 was not detected in *E. coli* Ag43^−ve^ MVs or their corresponding MV-depleted supernatant (Fig. 2A), indicating that Ag43 was associated only with purified Ag43^+ve^ MVs.

Having determined that Ag43 was associated with purified *E. coli* MVs, we next sought to visualize the presence of Ag43 on the surface of *E. coli* MVs using immunogold labeling and transmission electron microscopy (TEM; Fig. 2B through E). Examination of all MV samples by TEM revealed that no Ag43 was detected on the surface of Ag43^−ve^ MVs as expected (Fig. 2B), whereas Ag43 was detected on the surface of *E. coli* Ag43^+ve^ MVs as indicated by multiple gold particles bound to their surface (Fig. 2C). In addition, minimal non-specific binding of the gold particles to MVs was observed (Fig. 2D and E). Overall, these findings demonstrate that MVs produced by Ag43^+ve^ *E. coli* contain the passenger domain of Ag43 on their surface. Given that surface translocation of autotransporters such as Ag43 is associated with their correct folding and therefore, their functionality, this indicates that Ag43 on the MV surface has been translocated and is functionally active to promote bacterial interactions (43, 44). Indeed, an observation from our TEM experiments was that Ag43^+ve^ MVs consistently appeared to clump together (Fig. 2C and E), unlike those derived from Ag43^−ve^ *E. coli* (Fig. 2B and D), suggesting that MV-associated Ag43 may be functionally active, which we next investigated further.

### Ag43^+ve^ MVs interact with Ag43^+ve^ *E. coli* bacteria

**Fig 3:**
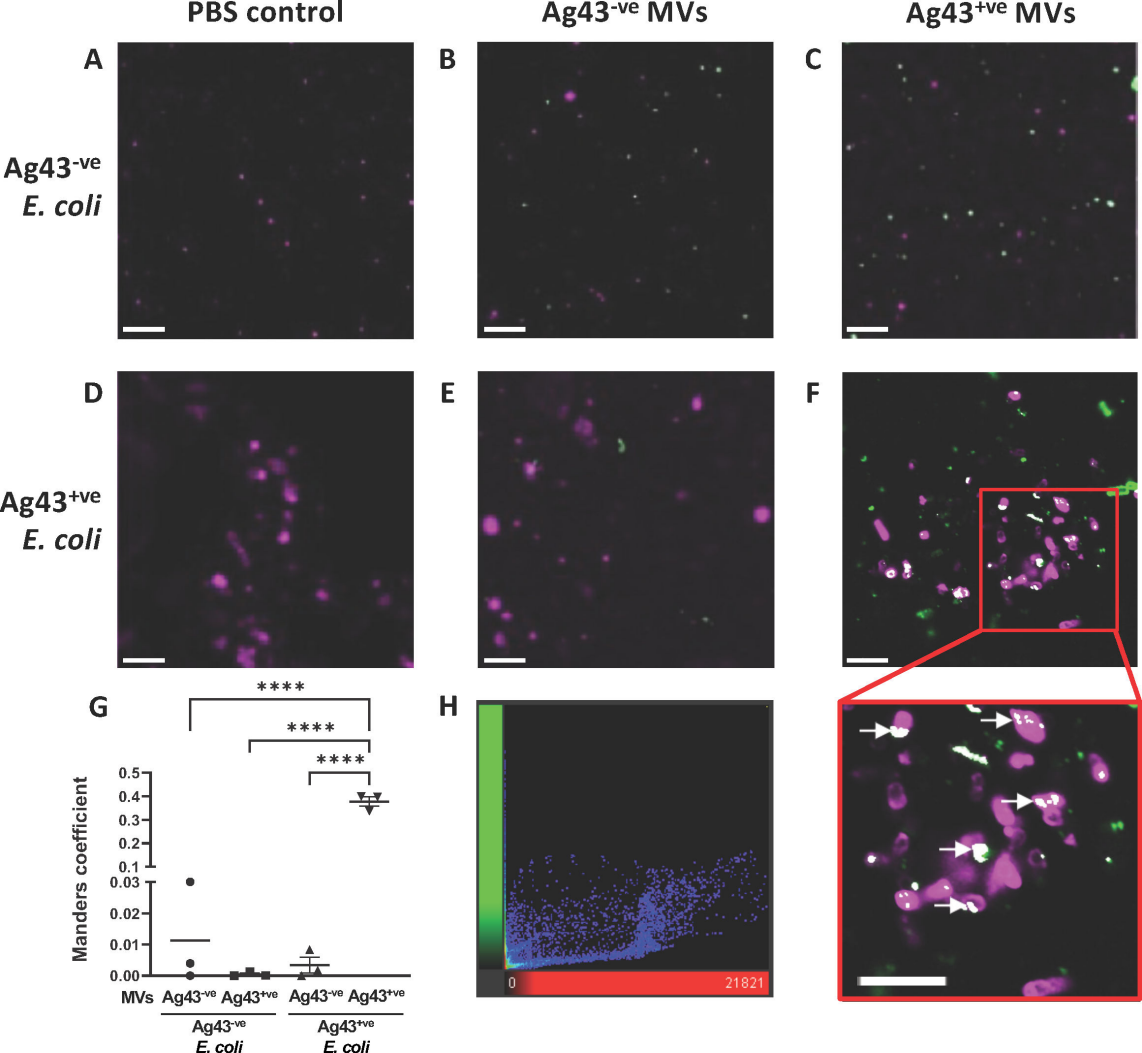
Ag43 positive MVs interact with *E. coli* expressing Ag43. Interactions between *E. coli* HEHA16 bacteria and purified MVs were observed using confocal microscopy. Ag43^−ve^ or Ag43^+ve^ *E. coli* bacteria were labeled with DiI (shown as magenta) and Ag43^−ve^ or Ag43^+ve^ MVs were labeled with DiO (green) lipophilic stains and incubated together in various combinations as indicated for 3 h. (**A**) Ag43^−ve^ *E. coli* with PBS, (**B**) Ag43^−ve^ *E. coli* with Ag43^−ve^ MVs, (**C**) Ag43^−ve^ *E. coli* with Ag43^+ve^ MVs, (**D**) Ag43^+ve^ *E. coli* with PBS, (**E**) Ag43^+ve^ *E. coli* with Ag43^−ve^ MVs, and (**F**) Ag43^+ve^ *E. coli* with Ag43^+ve^ MVs. Colocalization is shown as white and indicated by the white arrows. Scale bar = 5 µm. Images are representative of three biological replicates. (**G**) Colocalization of *E. coli* and MVs as quantified by Manders coefficient between combinations of DiI labeled Ag43^+ve^ or Ag43^−ve^ *E. coli* bacteria and their DiO labeled MVs. Data are represented as the mean ± SEM of three biological replicates. ****P < 0.0001. One-way ANOVA with Tukey’s multiple comparisons test. (**H**) Cytofluorogram showing colocalization between DiI labeled Ag43^+ve^*E. coli* and DiO labeled Ag43^+ve^ MVs. Data are representative of three biological replicates with ≥3 fields of view per biological replicate.

Ag43 can facilitate binding between *E. coli* cells, allowing for bacterial aggregation and the formation of biofilms (30, 31, 45). As the contribution of MVs containing Ag43 in facilitating bacterial self-adhesion and biofilm formation remains unknown, we next investigated if Ag43^+ve^ MVs could associate with and bind to Ag43^+ve^ *E. coli* bacteria. To do this, DiO (green) fluorescently labeled Ag43^−ve^ or Ag43^+ve^ MVs were added to DiI (shown as magenta) fluorescently labeled *E. coli* Ag43^−ve^ or Ag43^+ve^ bacteria, and interactions between bacteria and MVs were observed using confocal microscopy (Fig. 3). Initially, we observed that Ag43^+ve^ MVs were able to self-aggregate, suggesting that Ag43 on the surface of MVs was functional. We found that no aggregation was observed when DiI-labeled Ag43^−ve^ *E. coli* bacteria were incubated in the presence of either DiO-labeled Ag43^−ve^ MVs, Ag43^+ve^ MVs, or PBS as a control (Fig. 3A through C; Fig. S1A and B) indicating that significant interactions between *E. coli* bacteria and MVs did not occur if *E. coli* bacteria lacked Ag43 expression. In addition, incubation of DiI-labeled Ag43^+ve^ *E. coli* bacteria with either DiO-labeled Ag43^−ve^ MVs or PBS alone as a control resulted in aggregation of Ag43^+ve^ *E. coli* bacteria; however, no interactions with Ag43^−ve^ MVs were observed (Fig. 3D and E; Fig. S1C). In comparison, incubation of DiI-labeled Ag43^+ve^ *E. coli* bacteria with Ag43^+ve^ MVs resulted in colocalization of Ag43^+ve^ *E. coli* with Ag43^+ve^ MVs, indicating binding of MVs to bacteria resulted in aggregation (Fig. 3F, white arrows). Furthermore, we observed that Ag43^+ve^ MVs were also self aggregating, suggesting that Ag43 on the surface of MVs was functional. Analysis of colocalization using Mander’s colocalization coefficient revealed that Ag43^+ve^ *E. coli* and Ag43^+ve^ MVs colocalized together significantly more than any other combination of *E. coli* and MVs (Fig. 3G and H, *P* < 0.0001, Mander’s colocalization coefficient of 0.38 ± 0.02). Overall, our results identified that Ag43^+ve^ MVs were capable of binding and interacting with Ag43-expressing *E. coli* bacteria, which was mediated by the presence of Ag43 on both the bacterial and MV surface.

### Ag43^+ve^ MVs enhance the formation of *E. coli* biofilms

**Fig 4:**
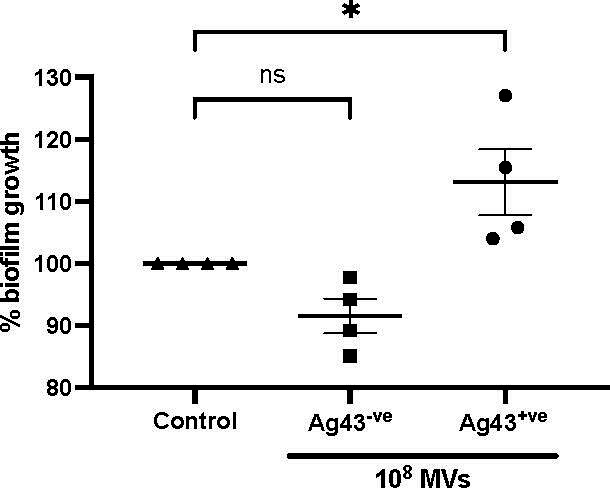
Ag43^+ve^ MVs enhance the formation of *E. coli* bacterial biofilms. *E. coli* MS528 was grown for 44 h in the presence of either 10^8^ Ag43^−ve^ MVs (squares), 10^8^ Ag43^+ve^ MVs (circles), or PBS as a control (Control; triangles) to produce biofilms. The amount of biofilm formation was determined using a crystal violet biofilm assay. Biofilm formation was normalized to Ag43^+ve^ *E. coli* biofilm grown in the presence of PBS and represented as a percentage of biofilm growth. Data are represented as the mean ± SEM of four biological replicates. One-way ANOVA with Dunnett’s multiple comparison test, *P < 0.05. ns, not significant.

**Fig 5:**
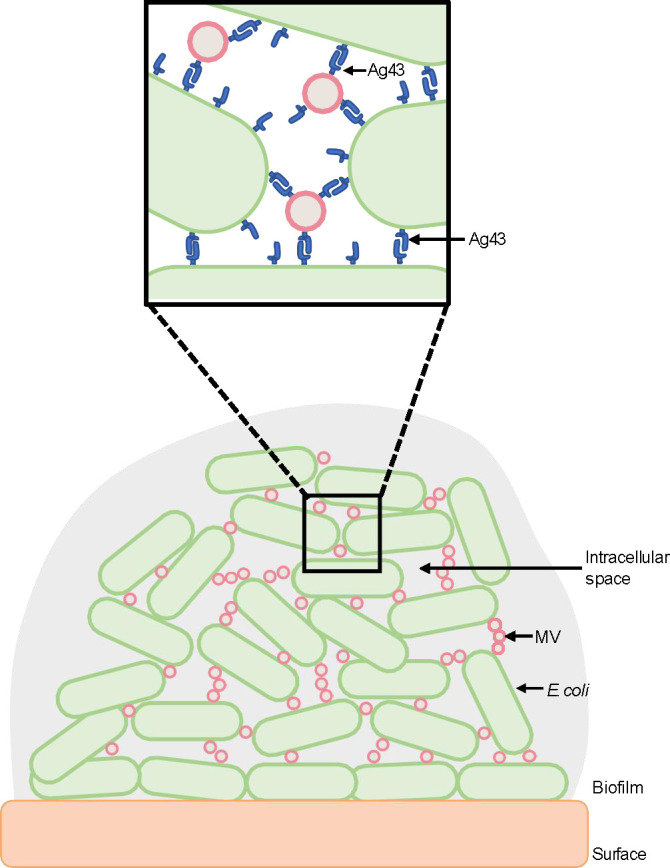
Model depicting interactions between *E. coli* bacteria and MVs facilitated by Ag43. Interactions between nearby *E. coli* bacteria are facilitated by the binding of Ag43 on the bacterial surface in an Ag43-Ag43 manner. In this study, we determined that the addition of Ag43^+ve^ MVs increased biofilm production by Ag43-expressing *E. coli* bacteria. Therefore, the presence of Ag43 on the surface of *E. coli* MVs may mediate greater number of interactions between Ag43 positive *E. coli* bacteria. Thus, Ag43-containing MVs may enhance biofilm formation by increasing the overall bacterial biofilm biomass.

Expression of Ag43 by bacteria has previously been shown to promote and drive the formation of *E. coli* and multi-species biofilms (25, 45). Therefore, as Ag43^+ve^ MVs could interact with Ag43^+ve^ bacteria, we next investigated if the addition of Ag43^+ve^ MVs to *E. coli* could enhance biofilm development. Biofilm assays were performed using Ag43^+ve^ *E. coli* MS528 *Δfim::kan Δagn43* bacteria harboring the pBAD/MycHisA::*agn43* plasmid, as *E. coli* HEHA16 is not capable of forming biofilms *in vitro* (34, 46). To do this, Ag43^+ve^ *E. coli* MS528 bacteria were incubated in the presence of an equivalent number of either Ag43^−ve^ MS528 MVs, Ag43^+ve^ MS528 MVs, or PBS as a control and allowed to develop a biofilm for 44 h. Incubation of *E. coli* Ag43^+ve^ bacteria alone resulted in the formation of biofilms, represented as 100% biofilm growth (Fig. 4). The addition of 10^8^ Ag43^+ve^ MVs to Ag43^+ve^ *E. coli* bacteria significantly enhanced biofilm development, suggesting a contribution of Ag43-expressing MVs to promoting biofilm development (*P* < 0.05; Fig. 4). In comparison, the addition of 10^8^ Ag43^−ve^ MVs to *E. coli* bacteria did not significantly affect *E. coli* biofilm formation (Fig. 4). Overall, our results demonstrate that the presence of Ag43^+ve^ MVs enhances biofilm development, by mediating interactions between Ag43^+ve^ bacteria and increasing overall bacterial biomass to promote biofilm formation (Fig. 5).

## DISCUSSION

Autotransporter adhesins that promote biofilm formation are a highly abundant and widespread group of surface proteins found throughout Gram-negative bacteria (47). Their expression on the cell surface of bacteria makes it plausible that MVs may also display autotransporters on their surface and potentially contribute to biofilm formation. Although the association of autotransporters with MVs has been determined using proteomic studies, the surface location and functional activity of autotransporters expressed on MVs have not been definitively determined. For example, proteomics analysis of crude, non-purified extraintestinal pathogenic *E. coli* MVs identified the presence of putative autotransporters associated with MVs (48); however, the surface localization of these autotransporters could not be adequately determined as these MVs were not subjected to purification prior to analysis. More recently, proteomic analyses identified the presence of Ag43 precursor proteins associated with probiotic *E. coli* MVs (42) and full-length Ag43 protein within detergent-derived uropathogenic *E. coli* MVs (41). However, to the best of our knowledge, Ag43 has not been identified in purified MVs naturally released by *E. coli*. Furthermore, MVs have previously been shown to facilitate the development of biofilm formation (11, 12), but the exact mechanisms by which this occurs for *E. coli*, in addition to the contribution of autotransporters to this process, has not been determined. Therefore, in this work, we sought to investigate the presence of functional active autotransporters on the surface of MVs, by focusing on Antigen 43, an *E. coli* autotransporter associated with bacterial cell aggregation and biofilm formation.

Previous research has shown that type 1 fimbriae and autotransporters such as Ag43 undergo phase variation and are inversely regulated, playing complementary, sequential roles during infection. Type 1 fimbriae mediate long-distance initial attachment and colonization, while autotransporters like Ag43 assume a crucial role in later stages by promoting close host cell adhesion, bacterial aggregation, and biofilm formation. In this study, we aimed to mimic infection conditions by employing a mutant *E. coli* strain lacking type 1 fimbriae and Ag43. A plasmid encoding Ag43 was introduced to control its expression. To explore the relationship between Ag43 expression and MV production, we purified MVs from *E. coli* strains harboring either a plasmid encoding for the inducible overexpression of Ag43 (Ag43^+ve^), or containing an empty plasmid as a negative control (Ag43^−ve^) (34, 36, 46). We found that Ag43^−ve^ and Ag43^+ve^ MVs were similar in morphology; however, expression of Ag43 in *E. coli* resulted in a significant increase in the number of MVs produced compared to the Ag43^−ve^ *E. coli* strain (Fig. 1). This increase in MV yield may be due to the stress caused by the overexpression of the Ag43 protein into the outer membrane, where *E. coli* may compensate for this by releasing Ag43 into MVs, as alterations in outer membrane proteins affects MV production in other bacterial species (49–51). In addition to changes in the number of MVs released, we also observed a shift in the size of MVs produced, as Ag43^+ve^ *E. coli* MVs were significantly smaller in size than Ag43^−ve^ MVs (Fig. 1E and F) which may be due to increased MV production by *E. coli* overexpressing Ag43. Previous reports have identified changes in the size of MVs released by bacteria grown in conditions of stress, however, whether the size of MVs becomes smaller or larger compared to control MVs depends on the bacterial species from which MVs are isolated (49, 52, 53).

The association of Ag43 with MVs released from purified Ag43^+ve^ *E. coli* was confirmed using Western blot analysis (Fig. 2A). The 50 kDa Ag43 α-domain was found with both MVs and in MV depleted supernatants, suggesting a gradual release of Ag43 into the environment, similar to what occurs when Ag43 is expressed on the *E. coli* cell surface (40, 54). 35Importantly, we showed the presence of Ag43 on the surface of MVs by immunogold staining (Fig. 2B through E). Taken together, our results identified that naturally released *E. coli* MVs contain Ag43, both consistent with and extending previous studies that identified autotransporters, such as Antigen 43, associated with MVs isolated from various strains of *E. coli* including detergent derived MVs (41, 42, 48).

Further analysis of Ag43^+ve^ MVs using a previously established confocal microscopy technique (36) revealed that they bind to Ag43^+ve^ *E. coli* bacteria to promote aggregation. This binding was found to be dependent on the presence of Ag43 on both *E. coli* and MVs, as there were no interactions observed when either Ag43^−ve^ *E. coli* or Ag43^−ve^ MVs were used (Fig. 3A through F). This is consistent with the well-known Ag43-Ag43 molecular mechanisms of self-association that results in *E. coli* aggregation and biofilm formation (36).

The aggregation and clumping of *E. coli* and other bacteria is known to promote the formation of biofilms (47). The expression of Ag43 is recognized to facilitate biofilm growth (34, 55), as has the addition of exogenous MVs to bacterial biofilms (11, 12); thus, it can be hypothesized that Ag43 on the surface of *E. coli* MVs could interact with *E. coli* bacteria and further promote the formation of biofilms. Our results showed that the addition of Ag43^+ve^ MVs promoted biofilm formation of Ag43^+ve^ *E. coli* to levels greater than those observed for the formation of biofilms by Ag43^+ve^ *E. coli* in the absence of MVs. This increase in biofilm formation was dependent on MV surface-associated Ag43, as the addition of MVs derived from Ag43^−ve^ *E. coli* did not enhance biofilm growth. This further confirms the interaction between Ag43 expressed by both MVs and bacteria and reveals that MVs seem to optimize bacteria-bacteria contacts, possibly by occupying intercellular spaces and enhancing the network of interactions that keep cells connected within a biofilm (Fig. 5). As Ag43 is a phase variable protein (24, 56), turning off Ag43 expression and therefore producing Ag43^−ve^ MVs may be a mechanism to catalyze the release of *E. coli* from biofilms to facilitate their spread.

Additionally, autotransporters have been identified as having a role in promoting adherence to host cells (57). Specifically, the expression of autotransporters present in *B. pertussis* MVs on the surface of *E. coli* bacteria resulted in increased adhesion of *E. coli* bacteria to epithelial A549 cells (58). Collectively, these studies highlight the contribution of MV-associated autotransporters in facilitating binding to host cell surfaces and modulating immunity in the host. The ability of autotransporter-expressing MVs to enhance bacterial colonization of host cell surfaces to promote pathogenesis remains to be determined.

Overall, this work advances our understanding of the relationship between MVs and highly prevalent autotransporters, including the effects of autotransporter-containing MVs on biofilm formation. Given that biofilms are associated with most types of bacterial infections and frequently lead to chronic and life-threatening conditions, it is of utmost importance that we can understand the processes leading to the formation of these recalcitrant bacterial structures. As bacterial biofilms provide increased resistance to antibiotic treatment (59–61), these processes also represent valid targets for the development of new antimicrobials that can target resistant bacterial biofilms (62). Our findings show that the ability of MVs to integrate biofilm-promoting proteins, such as Ag43, makes them important contributors to biofilm formation. It is highly likely that these features are widespread throughout other bacteria, with MVs integrating other self-associating autotransporters and groups of biofilm-promoting autotransporters. We also now know that biofilm-promoting autotransporters such as Ag43 are able to interact with homologs in autotransporters in other pathogenic strains (37, 38) and potentially other species, which would make MVs containing autotransporters important for regulating multi-species biofilm communities.

## MATERIALS AND METHODS

### Bacterial culture conditions

The following *E. coli* strains were used throughout this study: *E. coli* HEHA16 containing either the plasmid pBAD/MycHisA (Ag43^−ve^) or pBAD/MycHisA::*agn43* (Ag43^+ve^) (34) and *E. coli* MS582 containing either the plasmid pBAD/MycHisA (Ag43^−ve^) or pBAD/MycHisA::*agn43* (Ag43^+ve^) (25). All *E. coli* strains were routinely cultured on Luria Bertani Agar (LB Agar), consisting of Tryptone (Oxoid, USA), Yeast Extract (Oxoid, USA), Sodium Chloride (ChemSupply, Australia), and Agar Base No. 2 (Oxoid, USA) or LB broth, supplemented with the addition of 100 µg/mL ampicillin (Fisher BioReagents, USA) for the maintenance of the pBAD/MycHisA plasmid or 100 µg/mL ampicillin and 50 µg/mL kanamycin (Sigma, USA) for the maintenance of the pBAD/MycHisA::*agn43* plasmid as previously described (32). The expression of Ag43 was induced by the addition of 0.2% L-arabinose (vol/vol; Sigma, USA). For MV production, all *E. coli* strains were grown aerobically in 10 mL of LB broth supplemented with the appropriate antibiotics overnight and then diluted to a starting density of 0.05 (OD_600nm_) in LB broth with no antibiotic selection, and incubated at 37°C, shaking at 200 rpm for 2 h or until the OD_600nm_ reached 0.1–0.2. The expression of Ag43 was then induced with the addition of 0.2% L-arabinose (vol/vol), and cultures were incubated for a further 7 h at 37°C, shaking at 200 rpm.

### MV isolation and purification

MVs were isolated and purified using our established methods (63, 64). Briefly, 600 mL of *E. coli* cultured for 7 h was used to prepare MVs. Bacteria were pelleted by centrifugation at 4,000 × *g* for 15 min at 4°C, the resulting supernatant was filtered through a 0.22 µm filter, and MVs were pelleted by ultracentrifugation (Hitachi Ultracentrifuge CP100NX, Japan) at 100, 000 × *g* for 2 hr at 4°C.

*E. coli* MVs were further purified to remove contaminants using an iodixanol (OptiPrep; Sigma-Aldrich, USA) density gradient (63, 64). Briefly, MVs were resuspended in 45% (vol/vol) OptiPrep (1.1 mL) and underlaid in a discontinuous OptiPrep density gradient, consisting of 20%, 25%, 30%, 35%, and 40% OptiPrep solution. The discontinuous OptiPrep gradient was centrifuged at 100,000 × *g* for 16 h at 4°C. A total of 12 1 mL fractions were collected, with MVs being contained within fractions 2 to 10. MV fractions 2 to 10 were pooled and washed twice using Dulbecco’s phosphate-buffered saline (DPBS; Gibco, USA) by centrifugation at 100,000 × *g* for 2 h at 4°C. The resulting washed MV pellet was resuspended in DPBS and stored at −80°C until required. Before experimental use, MV samples were resuspended using a vortex for approximately 30 s to avoid MV clumping.

### Transmission electron microscopy

Transmission electron microscopy (TEM) was performed as previously described (14, 52, 65). Carbon-coated 400 mesh copper grids (ProSciTech, Australia) were pre-treated with poly-l-lysine (Sigma, USA). MV samples were coated onto grids by placing grids onto a 7 µL droplet of MVs for 10 min. Samples were then fixed using 1% (wt/vol) glutaraldehyde (Sigma, USA) diluted in PBS for 5 min, subsequently stained using 2% (wt/vol) uranyl acetate (ProSciTech, Australia) pH 7.0 for 5 min, and then coated with 2% (wt/vol) methyl-cellulose (Sigma, USA) in 0.4% uranyl acetate pH 4.0 for 10 min. Samples were air-dried and imaged using a JEOL JEM-2010 transmission electron microscope (JEOL, Japan) operated at 200 kV fitted with a Valeta 4 MP CCD camera (Emsis, Germany).

Immunogold labeling was performed as previously (4). Briefly, MVs on carbon-coated mesh copper grids were incubated with a rabbit polyclonal serum against Ag43a [1:3,000 (36, 37)] for 40 min followed by a goat anti-rabbit secondary antibody (1:5,000; ThermoFisher Scientific, USA) for 30 min and protein-A-gold (1:50; ProSciTech, Australia) for 30 min. Grids were then fixed, stained, and imaged by TEM.

### Nanoparticle tracking analysis

MV size and concentration were determined using the nanoparticle tracking analyzer ZetaView basic PMX-120 NTA (Particle Metrix, Germany), as previously (65). MVs were diluted to an appropriate concentration in DPBS in a final volume of 1 mL. Instrument calibration was performed using 102 nm polystyrene beads (ThermoFisher, USA) according to the manufacturer’s guidelines. Measurements were performed using a 405 nm 68 mW laser and CMOS camera. Measurements were taken at 11 cell positions and captured 60 frames per position at 25°C with camera sensitivity 80, shutter speed 100, autofocus, and automatic scattering intensity. Data were analyzed using the ZetaView software version 8.05.12 SPI with the following parameters: maximum area: 1,000, minimum area: 5, maximum brightness: 255, minimum brightness: 30, and minimum trace length 15. The average of three biological replicates was plotted as particle size versus number of particles per mL using GraphPad Prism v9.4.1.

### Detection of Ag43 by Western blot

Ag43^−ve^ MVs, Ag43^+ve^ MVs, MV-depleted supernatant, and Ag43 positive control protein were separated using a 12.5% SDS-PAGE gel and reducing conditions and subsequently transferred onto a 0.45 µm polyvinylidene difluoride membrane (PVDF; Amersham Hybond, UK). The membrane was blocked using 5% (wt/vol) skim milk diluted in Tris-buffered saline with 0.05% tween (TBST) for 2 h and incubated with rabbit anti-Ag43 antibodies (36) [1:3,000, 1% (wt/vol) skim milk in TBST] at 4°C for 16 h. The membrane was subsequently washed using TBST and incubated with goat anti-rabbit IgG (H + L) HRP antibodies (Pierce, USA, 1:5,000) for 2 h at room temperature. Membranes were developed using Clarity Enhanced chemiluminescence (ECL) Western blotting substrate (Bio-Rad, USA) and imaged using the Amersham Imager 600 (Cytiva, USA).

### Visualization of MV and *E. coli* interactions using confocal microscopy

Interactions between MVs and bacteria were examined using a modification of a previously established technique (36) and visualized by confocal microscopy. Fluorescent staining of bacteria and MVs was performed using our established techniques (65, 66). In brief, *E. coli* grown to an OD_600nm_ of 1 were stained with the lipophilic DiI stain (1:100; Invitrogen, USA) for 30 min at 37°C protected from light and then washed three times with PBS. MVs (3 × 10^10^) were stained with the lipophilic DiO stain (1:100; Invitrogen, USA) and washed with PBS using an Ultra Centrifugal filter (10 kDa MWCO; Sigma, USA) to remove free dye. DiI-labeled bacteria at an OD_600nm_ of 1 were incubated with 3 × 10^10^ DiO-labeled MVs for 3 h at room temperature protected from light. Samples were fixed to glass using poly-l-lysine (Sigma, USA) and VectaShield Mounting Medium (Vector Laboratories, USA) and imaged using a Zeiss LSM 800 confocal microscope (Zeiss, Germany) using the 63× oil objective. Image processing and Mander’s colocalization analysis were performed using the Imaris x64 v9.5.0 (Bitplane, Switzerland).

### Biofilm formation assay

Biofilm formation assays were adapted from previously established techniques (25, 46). *E. coli* MS528 pBAD/MycHisA::*agn43* was grown in LB to an OD_600nm_ of 0.1 in the absence of l-arabinose and then diluted 1:50 in 1/5 tryptic soy broth (TSB) with 100 µg/mL of ampicillin and 0.2% l-arabinose. Biofilm assays were performed in a 96-well plate (Corning, USA) with each well consisting of 10^8^ MVs resuspended in 10 µL of PBS and 40 µL of diluted bacterial culture. Ag43^+ve^ *E. coli* MS528 was grown with PBS as a control. Biofilm assays were incubated at 28°C for 44 h statically. After incubation, biofilms were rinsed with PBS, fixed with methanol, and stained with 0.1% crystal violet. Biofilms were washed with PBS, and the remaining crystal violet was solubilized with 30% acetic acid. Absorbance was measured at 590 nm using a ClarioStar plate reader (BMG LABTECH, Germany).

### Statistical analysis

All statistical analyses were performed using GraphPad Prism v9.4.1. All data are presented as the mean ± SEM of biological replicates. Statistical analyses were performed using the unpaired Student’s *t*-test or one-way ANOVA with Dunnett’s multiple comparisons test as indicated. Differences were considered significant when *P < 0.05.

## SUPPLEMENTAL MATERIAL

Figure S1 spectrum.01890-24-s0001.pdf *E. coli* bacteria and MVs do not aggregate to one another without the presence of Ag43 on either the bacterial or MV surface.

ASM does not own the copyrights to Supplemental Material that may be linked to, or accessed through, an article. The authors have granted ASM a non-exclusive, world-wide license to publish the Supplemental Material files. Please contact the corresponding author directly for reuse.
