# Supplementary material for: Antigen 43 associated with Escherichia coli membrane vesicles contributes to bacterial cell association and biofilm formation
Source: Microbiol Spectr. 2025 Jan 22;13(3):e01890-24. doi: 10.1128/spectrum.01890-24 (PMC11878089; doi:10.1128/spectrum.01890-24)
Supplement: Figure S1 — E. coli bacteria and MVs do not aggregate to one another without the presence of Ag43 on either the bacterial or MV surface. [file spectrum.01890-24-s0001.pdf]

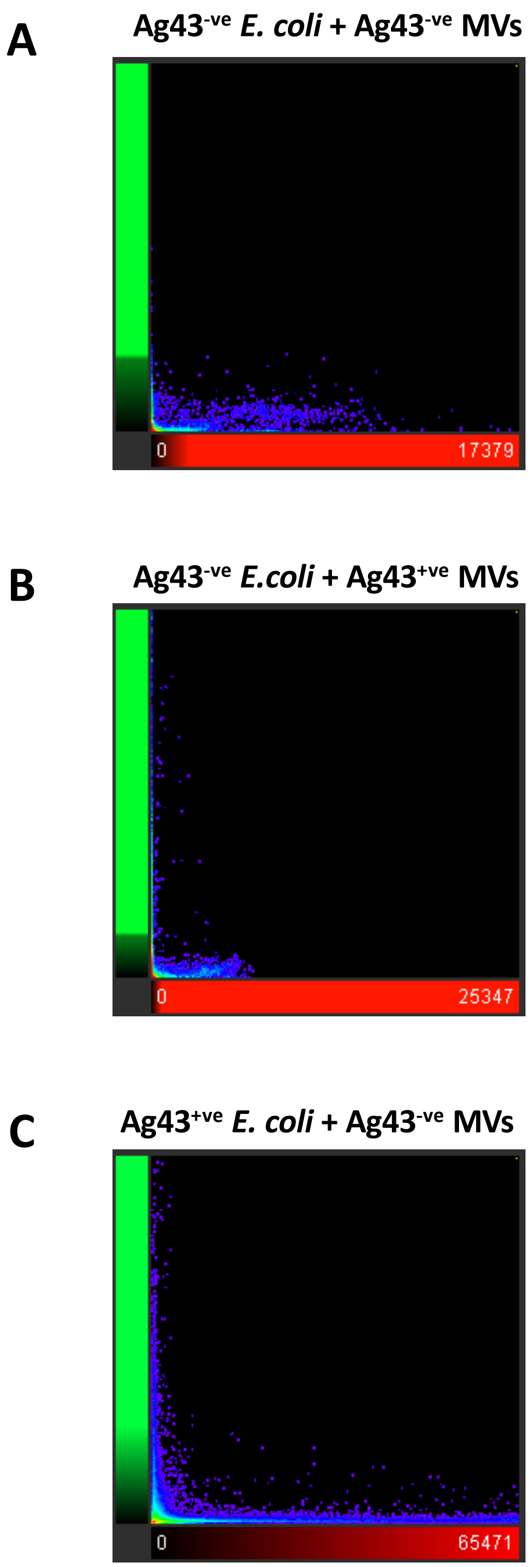

Supplemental Figure 1. ***E. coli*** **bacteria and MVs do not aggregate to one another without the presence of Ag43 on either the bacterial or MV surface.** Cytofluorograms showing no colocalization between **A)** DiI labeled Ag43^{-ve} *E. coli* incubated with DiO labeled Ag43^{-ve} MVs, **B)** DiI labeled Ag43^{-ve} *E. coli* incubated with DiO labeled Ag43^{+ve} MVs, and **C)** DiI labeled Ag43^{+ve} *E. coli* incubated with DiO labeled Ag43^{-ve} MVs. Data is representative of 3 biological replicates with ≥ 3 fields of view per biological replicate.
